# Optimal Control of HIV/AIDS in the Workplace in the Presence of Careless Individuals

**DOI:** 10.1155/2014/831506

**Published:** 2014-06-26

**Authors:** Baba Seidu, Oluwole D. Makinde

**Affiliations:** ^1^Applied Mathematics Department, Faculty of Mathematical Sciences, University for Development, Navrongo, Ghana; ^2^Faculty of Military Science, Stellenbosch University, Private Bag X2, Saldanha 7395, South Africa

## Abstract

A nonlinear dynamical system is proposed and qualitatively analyzed to study the dynamics of HIV/AIDS in the workplace. The disease-free equilibrium point of the model is shown to be locally asymptotically stable if the basic reproductive number, *ℛ*_0_, is less than unity and the model is shown to exhibit a unique endemic equilibrium when the basic reproductive number is greater than unity. It is shown that, in the absence of recruitment of infectives, the disease is eradicated when *ℛ*_0_ < 1, whiles the disease is shown to persist in the presence of recruitment of infected persons. The basic model is extended to include control efforts aimed at reducing infection, irresponsibility, and nonproductivity at the workplace. This leads to an optimal control problem which is qualitatively analyzed using Pontryagin's Maximum Principle (PMP). Numerical simulation of the resulting optimal control problem is carried out to gain quantitative insights into the implications of the model. The simulation reveals that a multifaceted approach to the fight against the disease is more effective than single control strategies.

## 1. Introduction

HIV/AIDS is one of the diseases that have claimed and continue to claim the lives of millions of people worldwide. Over the past three decades alone, HIV/AIDS has claimed the lives of more than 25 million people, most of whom were from Sub-Saharan Africa where 1 in every 20 adults is living with HIV. In 2011 alone, for example, about 34 million people, globally, were living with HIV/AIDS, about 23.5 million of them were from Sub-Saharan Africa and about 1.7 million people died from the disease globally [1]. The disease places so many burdens not only on families as some bread winners are lost but also on governments who have to spend millions of dollars in the purchase of antiretroviral drugs and on other intervention schemes. In 2011, for example, there was a total global expenditure of about US$16.8 billion in the fight against HIV/AIDS [1]. In his address of state of the nation this year, the president of Ghana spoke of the government's commitment to providing about 5 million Dollars to local pharmaceutical companies to help in the production of antiretroviral drugs in the country. It is these effects of the disease that call for continuous research into the prevention and control of the disease.

Mathematical models have played a major role in increasing our understanding of the dynamics of infectious diseases. Several models have been proposed to study the effects of some factors on the transmission dynamics of these infectious diseases including HIV/AIDS and to provide guidelines as to how the spread can be controlled. Among these models include those of Anderson et al. [2] who presented a preliminary study of the transmission dynamics of HIV by proposing a model to study the effects of various factors on the transmission of the disease, Stilianakis et al. [3]. who proposed and gave a detailed analysis of a dynamical model that describes the pathogenesis of HIV, and Tripathi et al. [4] who proposed a model to study the effects of screening of unaware infective on the transmission dynamics of HIV/AIDS. Several other models proposed to study dynamics of HIV/AIDS can be found in ([5–13], and the references therein).

“HIV/AIDS is a major threat to the world of work: it is affecting the most productive segment of the labour force and reducing earnings, and it is imposing huge costs on enterprises in all sectors through declining productivity, increasing labour costs and loss of skills and experience. In addition, HIV/AIDS is affecting fundamental rights at work, particularly with respect to discrimination and stigmatization aimed at workers and people living with and affected by HIV/AIDS. The epidemic and its impact strike hardest at vulnerable groups including women and children, thereby increasing existing gender inequalities and exacerbating the problem of child labour” [14]. Due to the effects of HIV/AIDS on firms, the International Labour Organization sees the field of work as a major stakeholder in the fight against the disease. The ILO envisages a world that sees HIV as a workplace issue like any other disease/sickness. It envisages a world of work that makes efforts to prevent discrimination in any form against people with HIV and also makes efforts to provide healthy work environments through social dialogue, prevention, and care and support for people with HIV. Dixon et al. [15] studied the impact of HIV/AIDS on Africa's economic development while [16] studied the impact of AIDs on developing economies. Not much research has been done in the study of epidemic models that consider the effect of HIV/AIDS on productivity and how the workplace can contribute to the fight against the disease. Okosun et al. [17] presented a dynamical model that studied the impact of susceptibles and infectives with different levels of productivity on the spread of HIV/AIDS at the workplace. They sought to determine the optimal levels of education, antiretroviral therapy that is required to optimally reduce the spread of the disease and increase productivity. In this paper, we present an extension of the model of Okosun et al. [17] to include susceptibles and infectives with different behaviors towards sex and with varying levels of productivity. Thus, we consider a dynamical system that incorporates the effects Careful-Productive Susceptibles, Careful-Non-Productive Susceptibles, Careless-Productive Susceptibles, Careless-Non-Productive Susceptibles, and similar groups of infectives on the transmission dynamics of HIV/AIDS at the workplace. We study the optimal levels of various intervention strategies needed to optimally reduce the spread of the disease and increase productivity. To do this, we modify our basic model to include various intervention strategies to obtain an optimal control problem which is analyzed qualitatively using the Pontryagin's Maximum principle. The resulting optimal control problem is also solved numerically to gain more insights into the implications of the interventions. The remainder of the paper is organized as follows. In Section 2, we present the mathematical model describing the dynamics of the disease and some basic properties of the model are also presented. The equilibrium states of the model and some implications are discussed in Section 3. In Section 4, we present a modification of the basic model into an optimal control problem and, finally, we present the results of the numerical simulations of the resulting optimal control problem in Section 5.

## 2. Formulation of the Model

**Figure 1:**
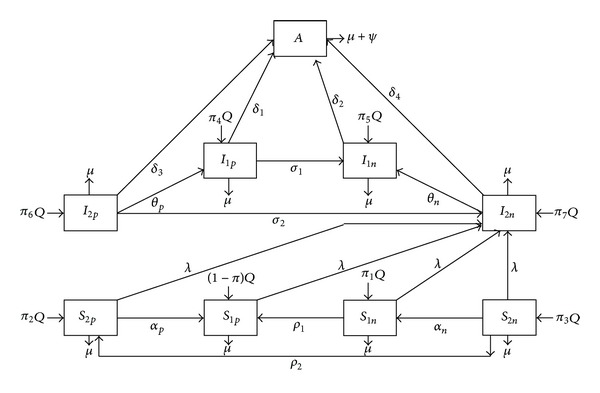
Flowchart of model (1).

In this section, we develop a deterministic model that describes the dynamics of HIV/AIDS in a homogeneously mixed workplace of population size *N*. The population is subdivided into nine (9) mutually-exclusive compartments, namely, Careful Productive Susceptibles, *S*_1*p*_; Careful Non-Productive Susceptibles, *S*_1*n*_; Careless-Productive Susceptibles, *S*_2*p*_; Careless-Non-Productive Susceptibles, *S*_2*n*_; Careful-Productive Infectives, *I*_1*p*_; Careful-Non-Productive Infectives, *I*_1*n*_; Careless-Productive Infectives, *I*_2*p*_; Careless-Non-Productive Infectives, *I*_2*n*_; and AIDS patients, *A*, so that we have *N* = *S*_1*p*_ + *S*_1*n*_ + *S*_2*p*_ + *S*_2*n*_ + *I*_1*p*_ + *I*_1*n*_ + *I*_2*p*_ + *I*_2*n*_ + *A*. The schematic diagram of the model is shown in Figure 1.

Our model assumes that there is a constant recruitment rate, *Q*, into the population with *π*_1_, *π*_2_, *π*_3_, *π*_4_, *π*_5_, *π*_6_, and *π*_7_ being the fractions of the respective subpopulations recruited into the population. Susceptible individuals acquire HIV through contact with infected ones with force of infection given by *λ* = *βc*[*I*_1*p*_ + *I*_1*n*_ + *τ*(*I*_2*p*_ + *I*_2*n*_)]/*N*, where *β* is the probability of infection per contact and *c* is the average number of sexual partners per unit time and *τ* is a modification parameter due to irresponsibility, which we assume is a factor that increases the chance of an infective transmitting the disease as they may tend to have a negative attitude towards protected sex. Newly infected susceptibles are assumed to be irresponsible and nonproductive as they are often unaware of their HIV status in the early stages and their productivity will reduce due to the infection. This is because, in the asymptomatic phase of the infection, infectives will often experience occasional fevers and general feeling of tiredness and non-feeling-well among others which can negatively impact the productivity. Due to the administration of highly active antiretroviral therapy (HAART), the responsible and irresponsible nonproductive infectives become responsible and irresponsible productives at the rates *σ*_1_ and *σ*_2_, respectively. Responsible and irresponsible nonproductive susceptibles become productive at the rates *ρ*_1_ and *ρ*_2_, respectively. Careful-Productive Infectives, Careful-Non-Productive Infectives, Careless-Productive Infectives, and Careful-Non-Productive Infectives develop AIDS at the rates *δ*_1_, *δ*_2_, *δ*_3_, and *δ*_4_, respectively. There is a positive change in behavior leading to Careless individuals (Productive Susceptibles, Nonproductive Susceptibles, Productive Infectives, and Nonproductive Infectives) becoming careful individuals (Respectively, Productive Susceptibles, Nonproductive Susceptibles, Productive Infectives, and Nonproductive Infectives) at rates *α*_*p*_, *α*_*n*_,*θ*_*p*_, and *θ*_*n*_, respectively. There is a natural death rate of *μ* for all individuals in all subgroups and *ψ* is the disease-induced death rate.

Putting the above formulations and assumption leads to the following set of ordinary differential equations representing the model describing the dynamics of HIV/AIDS at the workplace:

(1) dS1pdt=(1−∑k=17πk)QN+αpS2p+ρ1S1n−(λ+μ)S1p,dS1ndt=π1QN+αnS2n−(λ+ρ1+μ)S1n,dS2pdt=π2QN+ρ2S2n−(λ+αp+μ)S2p,dS2ndt=π3QN−(λ+αn+ρ2+μ)S2n,dI1pdt=π4QN+θpI2p+σ1I1n−(δ1+μ)I1p,dI1ndt=π5QN+θnI2n−(σ1+δ2+μ)I1n,dI2pdt=π6QN+σ2I2n−(θp+δ3+μ)I2p,dI2ndt=π7QN+λ(S1n+S2n+S1p+S2p)   −(σ2+θn+δ4+μ)I2n,dAdt=δ1I1p+δ2I1n+δ3I2p+δ4I2n−(ψ+μ)A.

By using *s*_1*p*_ = *S*_1*p*_/*N*, *s*_1*n*_ = *S*_1*n*_/*N*, *s*_2*p*_ = *S*_2*p*_/*N*, *s*_2*n*_ = *S*_2*n*_/*N*, *i*_1*p*_ = *I*_1*p*_/*N*, *i*_1*n*_ = *I*_1*n*_/*N*, *i*_2*p*_ = *I*_2*p*_/*N*, *i*_2*n*_ = *I*_2*n*_/*N*, and *a* = *A*/*N* and keeping *S*_1*p*_ = *s*_1*p*_,…, *I*_1*p*_ = *i*_1*p*_,…,  *A* = *a* for convenience, we have

(2) dS1pdt=(1−∑k=17πk)Q+αpS2p+ρ1S1n−(λ∗+μ)S1p,dS1ndt=π1Q+αnS2n−(λ∗+k1)S1n,dS2pdt=π2Q+ρ2S2n−(λ∗+k2)S2p,dS2ndt=π3Q−(λ∗+k3)S2n,dI1pdt=π4Q+θpI2p+σ1I1n−k4I1p,dI1ndt=π5Q+θnI2n−k5I1n,dI2pdt=π6Q+σ2I2n−k6I2p,dI2ndt=π7Q+λ∗(S1n+S2n+S1p+S2p)−k7I2n,dAdt=δ1I1p+δ2I1n+δ3I2p+δ4I2n−k8A,

where

(3) $$\begin{array}{c} \lambda_{*}=\beta c\left[I_{1p}+I_{1n}+\tau \left(I_{2p}+I_{2n}\right)\right],\\ k_{1}=\rho_{1}+\mu ,\;\;k_{2}=\alpha_{p}+\mu ,\;\;k_{3}=\alpha_{n}+\rho_{2}+\mu ,\\ k_{4}=\delta_{1}+\mu ,\;\;k_{5}=\sigma_{1}+\delta_{2}+\mu ,\;\;k_{6}=\theta_{p}+\delta_{3}+\mu ,\\ k_{7}=\sigma_{2}+\theta_{n}+\delta_{4}+\mu . \end{array}$$

In the next section, some basic facts about model (2) are presented.

### 2.1. Basic Properties of the Model

We show in this section that model (2) is reasonable both mathematically and biologically. This is achieved via the following theorems.

The model is epidemiologically feasible if the following theorem is true.

**Theorem 1 .**

Let *X*(*t*) = (*S*_1*p*_(*t*), *S*_1*n*_(*t*), *S*_2*p*_(*t*), *S*_2*n*_(*t*), *I*_1*p*_(*t*), *I*_1*n*_(*t*), *I*_2*p*_(*t*), *I*_2*n*_(*t*),…, *A*(*t*)). If the initial values of the model are nonnegative (i.e., *x*(0) > 0), then solutions of model (2) remain positive for all time *t* > 0.

In particular,  lim⁡_*t*→*∞*_⁡*Sup*⁡*N*(*t*) ≤ *Q*/*μ*.

#### 2.1.1. Positive Invariant Region of the Model

Let, *X*(*t*) = (*S*_1*p*_(*t*), *S*_1*n*_(*t*), *S*_2*p*_(*t*), *S*_2*n*_(*t*), *I*_1*p*_(*t*), *I*_1*n*_(*t*), *I*_2*p*_(*t*), *I*_2*n*_(*t*),  …, *A*(*t*)). We shall analyze model (2) in the domain

(4) $$\begin{array}{c} D=\left\{X\in R_{+}^{9}:\overset{9}{\underset{k=1}{\sum }}X_{k}\left(t\right)\leq \frac{Q}{\mu }\right\}. \end{array}$$

The region, *D*, can be shown to be positively invariant (i.e., solutions in *D* will always remain in *D*).

**Theorem 2 .**

The region *D* is positively invariant for model (2) with initial conditions in **R**_+_^9^.

**Proof**

Let

(5) $$\begin{array}{c} N=S_{1p}+S_{1n}+S_{2p}+S_{2n}+I_{1p}+I_{1n}+I_{2p}+I_{2n}+A. \end{array}$$

Then, adding all equations of model (2) we have

(6) dNdt=Q−μ(S1p+S1n+S2p+S2n+I1p+I1n+I2p      +I2n+A)−ψA=Q−μN−ψA.

Thus, d*N*/d*t* ≤ *Q* − *μN*.

A standard comparison theorem [18] can be used to prove that *N*(*t*) ≤ *N*(0)*e* ^−*μt*^ + (*Q*/*μ*)(1 − *e* ^−*μt*^).

In particular, if *N*(0) ≤ *Q*/*μ*, then *N*(*t*) ≤ *Q*/*μ* as required.

This shows that the region *D* is positively invariant and that the dynamics of the model can be sufficiently studied in *D* inside which the model is considered to be epidemiologically and mathematically well posed [19]. This means that all solutions of the model starting in *D* will remain in *D* for all time, *t* > 0.

## 3. Equilibrium Points of the Model

The model exhibits two equilibrium points, namely, the disease-free equilibrium point, *E*_0_, and the endemic equilibrium point, E*.

### 3.1. The Disease-Free Equilibrium

The disease-free equilibrium point exists in the absence of the disease and is given by

(7) $$\begin{array}{c} E_{0}=\left(S_{1p}^{0},S_{1n}^{0},S_{2p}^{0},S_{2n}^{0},0,0,0,0,0\right), \end{array}$$

where

(8) S1p0=(1−π1−π2−π3)Qμ    +Q{ρ1k2k3π1+αpk1k3π2+[ρ1αnk2+ρ2αpk1]π3}μk1k2k3,S1n0=Q[k3π1+αnπ3]k1k3,S2p0=Q[k3π2+ρ2π3]k2k3,S2n0=π3Qk3.

#### 3.1.1. Basic Reproduction Number

We use the next generation matrix method of [20] to calculate the basic reproduction number,  *ℛ*_0_. The transmission and transition matrices are, respectively, given by

(9) $$\begin{array}{c} F=\left[\begin{array}{cccc} 0 & 0 & 0 & 0\\ 0 & 0 & 0 & 0\\ 0 & 0 & 0 & 0\\ \beta cS^{0} & \beta cS^{0} & \tau \beta cS^{0} & \tau \beta cS^{0} \end{array}\right], \end{array}$$

with *S* ^0^ = *S*_1*p*_^0^ + *S*_1*n*_^0^ + *S*_2*p*_^0^ + *S*_2*n*_^0^ and

(10) $$\begin{array}{c} V=\left[\begin{array}{cccc} k_{4} & -\sigma_{1} & -\theta_{p} & 0\\ 0 & k_{5} & 0 & -\theta_{n}\\ 0 & 0 & k_{6} & -\sigma_{2}\\ 0 & 0 & 0 & k_{7} \end{array}\right]. \end{array}$$

van den Driessche and Watmough [20] defined the basic reproduction number, *ℛ*_0_, as the largest eigenvalue of the matrix *FV* ^−1^.

Consider

(11) $$\begin{array}{c} R_{0}=\frac{\beta cQ\left(k_{5}\sigma_{2}\theta_{p}+k_{6}\theta_{n}\left(k_{4}+\sigma_{1}\right)+\tau k_{4}k_{5}\left(\sigma_{2}+k_{6}\right)\right)}{\mu k_{4}k_{5}k_{6}k_{7}}. \end{array}$$

Using theorem (2) of [20], the following theorem is established.

**Theorem 3 .**

The disease-free equilibrium point, *E*_0_, of model (2) is locally asymptotically stable if *ℛ*_0_ < 1 and unstable if *ℛ*_0_ > 1.

The basic reproduction ratio is a threshold quantity that measures the average number of secondary infections caused by a single infected individual introduced into a completely susceptible population over its duration of infectivity [19, 21]. Epidemiologically, Theorem 3 implies that a small influx of infectives will not lead to an epidemic if *ℛ*_0_ < 1. The theorem also implies that HIV/AIDs can be eradicated when *ℛ*_0_ < 1 provided that the initial population sizes are within the region of attraction of the disease-free equilibrium point.

#### 3.1.2. Sensitivity Analysis of Model Parameters

In this section, the relative effects of the parameters that determine *ℛ*_0_ are presented. We use the normalized forward sensitivity index defined as follows.

**Definition 4 .**

Let  *ℛ*_0_ = *f*(*x*_1_, *x*_2_,…, *x*_*n*_). Then the normalized forward sensitivity index of *ℛ*_0_ relative to *x*_*i*_ is given by *Υ*_*x*_*i*__^*ℛ*_0_^ = (∂*ℛ*_0_/∂*x*_*i*_) × (*x*_*i*_/*ℛ*_0_).

Thus, *Υ*_*β*_^*ℛ*_0_^ = *Υ*_*Q*_^*ℛ*_0_^ = 1. Due to the complex nature of the resulting expressions, the numerical sensitivity indexes of the remaining parameters are presented in Table 2. These indexes are evaluated using the parameter values in Table 1. Quite a number of these parameter values are used mainly for the simulation purposes to illustrate the kind of response expected for the given parameter values and may not be correct epidemiologically.

The sensitivity indexes reflect the percentage change in the dependent variable (in this case *ℛ*_0_) as a result of a percentage change in the independent variable. Thus, a 10% increase (or decrease) in the transmission probability, *β*, leads to a 10% increase (or decrease) in the basic reproduction number, while a 10% increase (or decrease) in the rate of progression of the Productive Infectives into AIDS leads to 4.6% decrease (or increase) in the basic reproduction number. It is observed from Table 2 that the most sensitive parameter is *μ* followed by *Q*, *β*, and *c*, which are equally sensitive. Thus, these parameters should be those that can be used to control the spread of the disease.

### 3.2. The Endemic Equilibrium

In the presence of the infection, the system exhibits the endemic equilibrium point, E*, given by

(12) $$\begin{array}{c} E^{*}=\left(S_{1p}^{*},S_{1n}^{*},S_{2p}^{*},S_{2n}^{*},I_{1p}^{*},I_{1n}^{*},I_{2p}^{*},I_{2n}^{*},A^{*}\right), \end{array}$$

where

(13) S1p∗=(1−∑k=17πk)Qλ∗+μ+(Q{ρ1(λ∗+k2)(λ∗+k3)π1+αp(λ∗+k1)×(λ∗+k3)π2    +[ρ1αn(λ∗+k2)+ρ2αp(λ∗+k1)π3]})×((λ∗+μ)(λ∗+k1)(λ∗+k2)(λ∗+k3))−1,S1n∗=Q[(λ∗+k3)π1+αnπ3](λ∗+k1)(λ∗+k3),S2p∗=Q[(λ∗+k3)π2+ρ2π3](λ∗+k2)(λ∗+k3),S2n∗=π3Qλ∗+k3,I1p∗=(Q[(λ+μ)(π4k5k6+σ1π5k6+θpπ6k5)      +(k5σ2θp+k6σ1θ1)      ×(μπ7+λ(1−π4−π5−π6))])      ×((λ+μ)k4k5k6)−1,I1n∗=Q[π5(λ+μ)+θn[μπ7+λ(1−π4−π5−π6)]]k5(λ+μ),I2p∗=Q[k7(λ+μ)π6+σ2[μπ7+λ(1−π4−π5−π6)]]k6(λ+μ),I2n∗=Q[μπ7+λ∗(1−π4−π5−π6)]k7(λ∗+μ),A∗=δ1I1p∗+δ2I2p∗+δ3I1n∗+δ4I2n∗ψ+μ.

After some algebraic manipulations, it can be shown that *λ*_∗_ satisfies the fourth order polynomial

(14) $$\begin{array}{c} P\left(\lambda_{*}\right)=k_{4}k_{5}k_{6}k_{7}\lambda_{*}^{2}+\Gamma_{1}\lambda_{*}+\Gamma_{0}=0, \end{array}$$

where

(15) Γ1=μk4k5k6k7[1−R0(1−π4−π5−π6)] −βcQ[k7π4k5k6+k7(k4+σ1)k6π5      +(k7θpk5+τk4k5k7)π6],Γ0=−βcQμ[k7π4k5k6+k7(k4+σ1)k6π5     +(k7θpk5+τk4k5k7)π6]−μk4k5k6k7R0π7.

When there is no recruitment of infectives (i.e., *π*_4_,…, *π*_7_ = 0), we have Γ_0_ = 0, Γ_1_ = *μk*_4_ *k*_5_ *k*_6_ *k*_7_(1 − *ℛ*_0_) and, hence, the polynomial has two roots, namely, *λ*_∗_ = 0 which corresponds to the disease-free equilibrium and the other being *λ*_∗_ = *μ*(*ℛ*_0_ − 1) which is positive if and only if *ℛ*_0_ > 1. Thus, in the absence of recruitment of infectives, the endemic equilibrium point exists only when *ℛ*_0_ > 1 and is given by (*S*_1*p*_^∗1^, *S*_1*n*_^∗1^, *S*_2*p*_^∗1^, *S*_2*n*_^∗1^, *I*_1*p*_^∗1^, *I*_1*n*_^∗1^, *I*_2*p*_^∗1^, *I*_2*n*_^∗1^, *A* ^∗1^), where

(16) S1p∗1=(1−(π1+π2+π3))Q+αpS2n∗1+ρ1S1n∗1μR0,S1n∗1=Q((μ(R0−1)+k3)π1+αnπ3)(μ(R0−1)+k1)(μ(R0−1)+k3),S2p∗1=Q((μ(R0−1)+k3)π2+ρ2π3)(μ(R0−1)+k2)(μ(R0−1)+k3),S2n∗1=π3Qμ(R0−1)+k3,I1p∗1=Q[k5σ2θp+k6σ1θn]k4k5k6k7(1−1R0),I1n∗1=Qθnk5k7(1−1R0),I2p∗1=Qσ2k6k7(1−1R0),I2n∗1=Qk7(1−1R0),A∗1=Qψ+μ(δ1(k5σ2θp+k6σ1θn)k4k5k6k7        +δ2θnk5k7+δ3σ2k6k7+δ4k7)(1−1R0).

Thus, the following theorem is established.

**Theorem 5 .**

In the absence of recruitment of infectives:

- if *ℛ*_0_ < 1, model (2) has exactly one equilibrium point which is the disease-free equilibrium;
- if *ℛ*_0_ > 1, model (2) has two equilibria, namely, the disease-free equilibrium (8) and the endemic equilibrium point (16), coexisting.

By Theorem 5, a necessary and sufficient condition for eradication of the disease in the absence of recruitment of infectives is that the basic reproduction number, *ℛ*_0_, be less than unity.

**Theorem 6 .**

In the presence of recruitment of infectives, model (2) has a unique positive equilibrium irrespective of the sign of  Γ_1_. Thus, in the presence of recruitment of infectives, the model does not exhibit backward bifurcation.

Since all model parameters are nonnegative, then clearly Γ_0_ < 0 and, hence, the discriminant of the quadratic equation, Δ = Γ_1_^2^ − 4*k*_4_ *k*_5_ *k*_6_ *k*_7_Γ_0_, is positive. By the Descartes rule of signs, the polynomial has two real roots of opposite signs. Hence, the model has a unique endemic (positive) equilibrium irrespective of the sign of Γ_1_.

## 4. Extended Model with Controls

In this section, an optimal control problem is formulated by incorporating four intervention strategies into our basic model (2). The following interventions are incorporated into the basic model:

- *u*_1_ is the control effort aimed at reducing the infection of susceptible individuals;
- *u*_2_ is the control effort aimed at treating infected individuals;
- *u*_3_ is the control effort aimed at changing behavior. That is, *u*_3_ is the control effort aimed at making Careless Susceptibles (both Productive and Nonproductive) and Infectives (both Productive and Nonproductive) Careful;
- *u*_4_ is the control effort aimed at reducing nonproductivity at the workplace.

Thus, the basic model becomes

(17) dS1pdt=(1−∑k=17πk)Q+u3αpS2p+u4ρ1S1n    −(λ∗(1−u1)+μ)S1p,dS1ndt=π1Q+u3αnS2n−(λ∗(1−u1)+u4ρ1+μ)S1n,dS2pdt=π2Q+u4ρ2S2n−(λ∗(1−u1)+u3αp+μ)S2p,dS2ndt=π3Q−(λ∗(1−u1)+u3αn+u4ρ2+μ)S2n,dI1pdt=π4Q+u3θpI2p+u4σ1I1n−((1−u2)δ1+μ)I1p,dI1ndt=π5Q+u3θnI2n−(u4σ1+(1−u2)δ2+μ)I1n,dI2pdt=π6Q+u4σ2I2n−(u3θp+(1−u2)δ3+μ)I2p,dI2ndt=π7Q+λ∗(1−u1)(S1n+S2n+S1p+S2p)    −(u4σ2+u3θn+(1−u2)δ4+μ)I2n,dAdt=(1−u2)(δ1I1p+δ2I1n+δ3I2p+δ4I2n)    −(ψ+μ)A.

Our main aim in developing this extended model is to seek optimal levels of the intervention strategies needed to minimize the number of nonproductive workers and the cost of implementing the control strategies. We choose a functional *J* given by

(18) J=min⁡ui, i∈[1,4]⁡∫0T[a1S1n+a2S2n+a3I1n+a4I2n         +12(w1u12+w2u22+w3u32+w4u42)]dt,

where the *w*_*i*_s are positive weights which measure relative costs of implementing the respective intervention strategies over the period [0, *T*], whilst the terms *w*_*i*_ *u*_*i*_^2^/2 measure the cost of the intervention strategies. We chose a quadratic cost functional in line with several other literatures on models of epidemic control [22–25]. Thus, we seek an optimal control quadruple (*u*_1_*, *u*_2_*, *u*_3_*, *u*_4_*) such that

(19) $$\begin{array}{c} J\left(u_{1}^{*},u_{2}^{*},u_{3}^{*},u_{4}^{*}\right)=\min\left\{J\left(u_{1},u_{2},u_{3},u_{4}\right)\;|\;u_{i}\in U\right\}, \end{array}$$

where *U* = {(*u*_1_, *u*_2_, *u*_3_, *u*_4_) such that *u*_*i*_ are measurable with 0 ≤ *u*_*i*_(*t*) ≤ 1; ∀*t* ∈ [0, *T*]} is the set of admissible controls.

Pontryagin's Maximum Principle [26] provides the necessary condition for optimality of the controls. Using this principle, (17) and (18) are converted into a problem of minimizing, with respect to the controls *u*_*i*_s, the Hamiltonian *H* given by

(20) H=a1S1n+a2S2n+a3I1n+a4I2n   +12(w1u12+w2u22+w3u32+w4u42)   +λ1[(1−∑k=17πk)Q+u3αpS2p+u4ρ1S1n     −(λ∗(1−u1)+μ)S1p]   +λ2[π1Q+u3αnS2n−(λ∗(1−u1)+u4ρ1+μ)S1n]   +λ3[π2Q+u4ρ2S2n−(λ∗(1−u1)+u3αp+μ)S2p]   +λ4[π3Q−(λ∗(1−u1)+u3αn+u4ρ2+μ)S2n]   +λ5[π4Q+u3θpI2p+u4σ1I1n−((1−u2)δ1+μ)I1p]   +λ6[π5Q+u3θnI2n−(u4σ1+(1−u2)δ2+μ)I1n]   +λ7[π6Q+u4σ2I2n−(u3θp+(1−u2)δ3+μ)I2p]   +λ8[π7Q+λ∗(1−u1)(S1n+S2n+S1p+S2p)       −(u4σ2+u3θn+(1−u2)δ4+μ)I2n]   +λ9{(1−u2)[δ1I1p+δ2I1n+δ3I2p+δ4I2n]       −(ψ+μ)A}.

The *λ*_*i*_s, (*i* = 1,…, 9) are the adjoint variables or costate variables which determine the adjoint system, which together with the state system (17) describes the optimality system.

Pontryagin's Maximum principle [26] and the existence result for optimal control from [27] can be used to obtain the following proposition.

**Proposition 7 .**

The optimal control 4-tuple (*u*_1_*, *u*_2_*, *u*_3_*, *u*_4_*) minimizes the functional *J* if there exist adjoint variables *λ*_*i*_, *i* = 1,…, 9 that satisfy the adjoint system given by

(21) dλ1dt=βc(I1p+I1n+τ(I2p+I2n))(1−u1)(λ1−λ8)    +λ1μ,dλ2dt=−a1+(λ2−λ1)ρ1u4+λ2μ+λ∗(λ2−λ8)(1−u1),dλ3dt=(λ3−λ1)αpu3+λ∗(1−u1)(λ3−λ8)+λ3μ,dλ4dt=−a2+λ∗(1−u1)(λ4−λ8)    +(λ4−λ2)αnu3+(λ4−λ3)ρ2u4+λ4μ,

(22) dλ5dt=ξ+(λ5−λ9)δ1(1−u2)+λ5μ,dλ6dt=ξ−a3+(λ6−λ5)σ1u4+λ6μ    +(λ6−λ9)δ2(1−u2),dλ7dt=τξ+(λ7−λ5)θpu3+λ7μ+(λ7−λ9)δ3(1−u2),dλ8dt=τξ−a4+(λ8−λ6)θnu3+(λ8−λ7)σ2u4+λ8μ    +(λ8−λ9)δ4(1−u2),dλ9dt=λ9(ψ+μ).

With transversality conditions *λ*_*i*_(*T*) = 0, ∀*i* = 1,…, 9, where

(23) ξ=βc[λ1S1p+λ2S1n+λ3S2p+λ4S2n  −λ8(S1p+S1n+S2p+S2n)](1−u1).

Further more

(24) u1∗(t)=min⁡{1,max⁡{λ∗((λ8−λ1)S1p+(λ8−λ2)S1n+(λ8−λ3)S2p+(λ8−λ4)S2n)w1,0}},u2∗(t)=min⁡{1,max⁡{(λ9−λ5)δ1I1p+(λ9−λ6)δ2I1n+(λ9−λ8)δ4I2n+(λ9−λ7)δ3I2pw2,0}},u3∗(t)=min⁡{1,max⁡{(−λ1+λ3)S2pαp+(λ4−λ2)S2nαn+(λ7−λ5)θpI2p+(λ8−λ6)θnI2nw3,0}},u4∗(t)=min⁡{1,max⁡{(λ2−λ1)S1nρ1+(λ4−λ3)S2nρ2+(λ6−λ5)σ1I1p+(λ8−λ7)σ2I2pw4,0}}.

**Proof**

We obtain the existence of the optimal controls from [27, Corollary 4.1] due to the convexity of the integrand of the functional *J* with respect to the quadruple (*u*_1_, *u*_2_, *u*_3_, *u*_4_),* a prior* boundedness of the state solutions, and the* Lipschitz* property of the state system with respect to the state variables. Using Pontryagin's Maximum Principle, the adjoint or costate equations (21) are obtained by differentiating the Hamiltonian partially with respect to the state variables. Thus, we have

(25) dλ1dt=−∂H∂S1p,  dλ2dt=−∂H∂S1n,  dλ3dt=−∂H∂S2p,dλ4dt=−∂H∂S2n,  dλ5dt=−∂H∂I1p,  dλ6dt=−∂H∂I1n,dλ7dt=−∂H∂I2p,  dλ8dt=−∂H∂I2n,  dλ9dt=−∂H∂A,with  λi(T)=0  for  i=1,…,9.

Since the Hamiltonian is minimized at the optimal controls, the optimality conditions ∂*H*/∂*u*_*i*_ = 0 at *u*_*i*_ = *u*_i_* are met. These optimality conditions can be used to obtain expressions for *u*_i_*. By standard control arguments involving the bounds on the controls, (24) is obtained, concluding the proof.

## 5. Numerical Simulations

### 5.1. Methodology

The solution of the optimal control problem is obtained by solving the optimality system which consists of the state and adjoint systems (17) and (21), respectively. For computational illustration, the values of parameters in Table 1 were employed and the solution is obtained by using the following iterative scheme.

**Step 1 .**

Make a guess of the controls.

**Step 2 .**

Use the values of the controls together with the initial conditions to solve the state equations, using a forward numerical scheme.

**Step 3 .**

Using the current solution of the state system together with the transversality conditions, solve the adjoint equations using a backward numerical scheme. We use a backward scheme for the costate system because the transversality conditions are final time conditions.

**Step 4 .**

Update the controls using the characterizations in (24).

**Step 5 .**

Repeat Steps 2 to 4 until the values of the unknowns at the current iteration are very close to those of the previous iteration [28].

We note here that human resource departments could only be concerned with reducing nonproductivity or seek to reduce the effect of HIV or combine both efforts. To compare the effects of these options, we consider the following combinations of the controls.

 Strategy 1: Implementing all controls (i.e., *u*_1_ ≠ *u*_2_ ≠ *u*_3_ ≠ *u*_4_ ≠ 0)
 Strategy 2: Implementing the controls aimed at reducing infection and treating infected individuals (i.e., *u*_1_ ≠ *u*_2_ ≠ *u*_3_, *u*_4_ = 0)
 Strategy 3: Implementing only the control effort aimed at reducing Nonproductivity (i.e., *u*_1_ = *u*_2_ = *u*_3_, *u*_4_ ≠ 0)
 Strategy  4: Implementing only *u*_1_ and *u*_3_
 Strategy 5: Implementing only *u*_2_.

### 5.2. Results

In this section, we present the results of the numerical simulation of our optimal control problem by discussing the implications implementing the five intervention schemes above.

**Figure 2:**
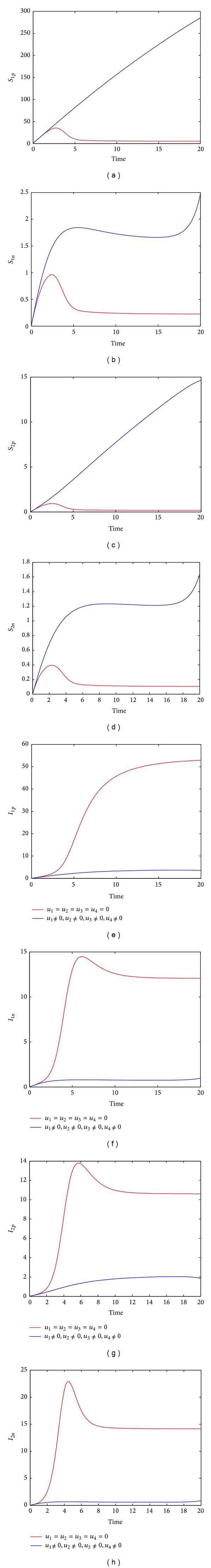
Simulations of Basic model (2) and the Optimal Control Problem (17) showing the effect of implementing all the intervention strategies on the dynamics of HIV/AIDS transmission.

**Figure 3:**
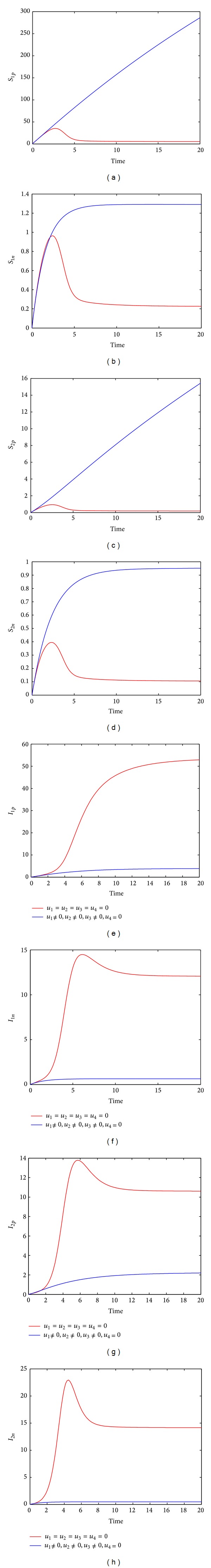
Simulations of the Optimal Control Problem (17) showing the effect of control Strategy 2 on the dynamics of HIV/AIDS transmission.

**Figure 4:**
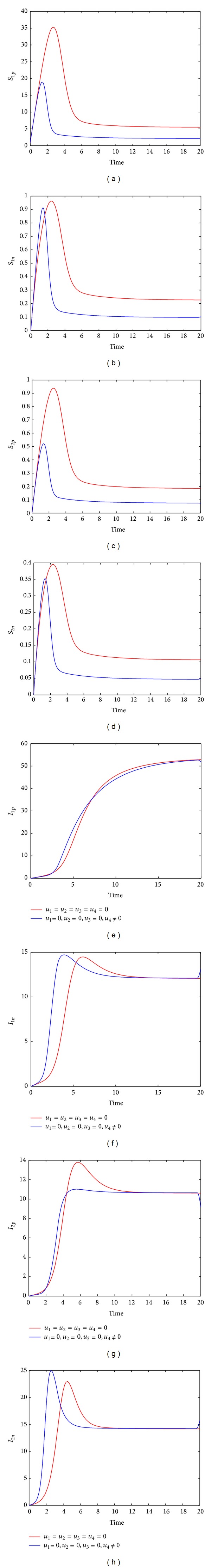
Simulations of the Optimal Control Problem (17) showing the effect of control Strategy 3 on the dynamics of HIV/AIDS transmission.

**Figure 5:**
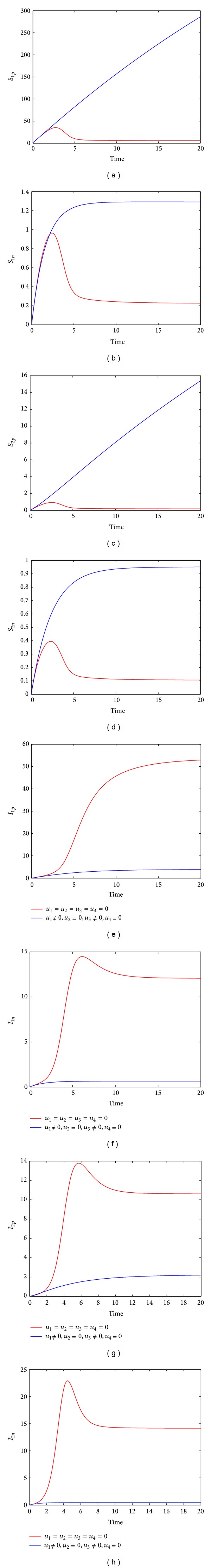
Simulations of the Optimal Control Problem (17) showing the effect of control Strategy 4 on the dynamics of HIV/AIDS transmission.

**Figure 6:**
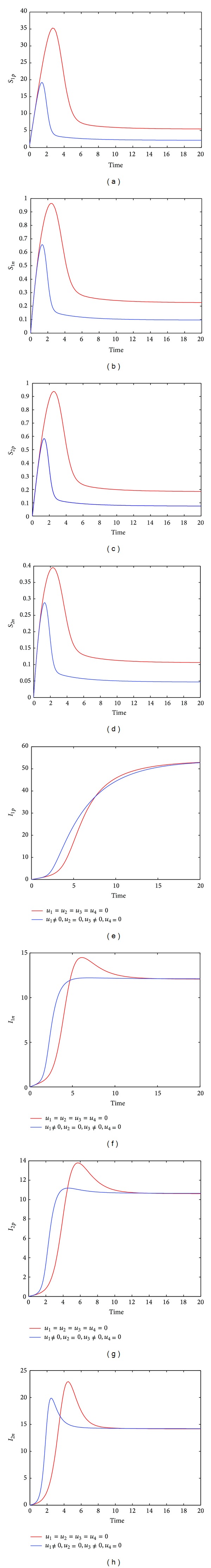
Simulations of the Optimal Control Problem (17) showing the effect of control Strategy 5 on the dynamics of HIV/AIDS transmission.

We examine the effects of applying the intervention schemes in each of the strategies. Thus, we aim to determine the optimal levels of the controls that will minimize the objective functional *J*. To observe the effects of the intervention strategies, we plot results from simulation of the uncontrolled model (2) and that from the controlled one (17) together in Figures 2 to 6. It is observed in Figures 2(a)–2(d), 3(a)–3(d), 4(a)–4(d), 5(a)–5(d) and 6(a)–6(d) that the number of susceptives remains higher for the controlled problem than for the uncontrolled problem. That means that each of the intervention strategies will lead to saving more people from being infected. It is also observed in Figures 2(e)–2(h), 3(e)–3(h), 4(e)–4(h), 5(e)–5(h) and 6(e)–6(h) that implementing the controls in each strategy will lead to a reduction in the number of people infected with the disease and also reduces the number of Nonproductive individuals.

**Figure 7:**
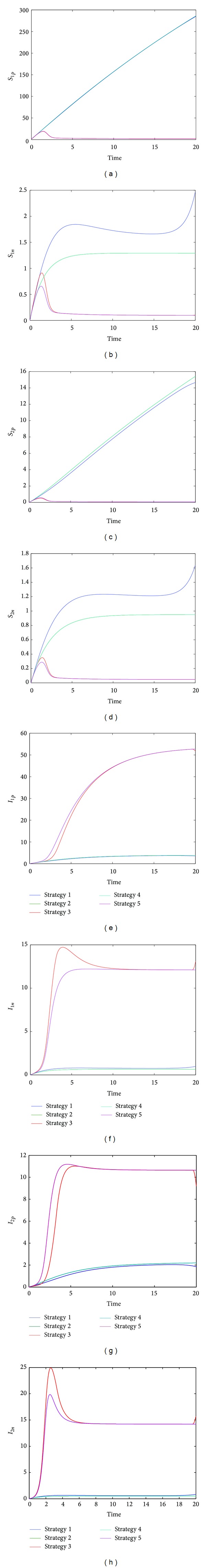
Simulations of the Optimal Control Problem (17) showing the effect of control Strategy 1 on the dynamics of HIV/AIDS transmission.

To compare the various strategies, we also plot the results of all the strategies on same graphs as in Figure 7. It is observed from the graphs in Figure 7 that the strategy that involves implementing all the controls leads to higher susceptible populations and lower infectives populations. This implies that the fight against HIV/AIDS should be multifaceted in order to achieve maximum benefits.

## 6. Conclusion

In this paper, a nonlinear dynamical model has been proposed to study the dynamics of HIV/AIDS in the workplace. The model assumes that there is no discrimination against people living with HIV and that, thus, allows for recruitment of both susceptible and infected individuals by the human resource department. Disease-free and endemic equilibrium states are shown to exist for certain parameter values of the model.

It is shown that the model cannot have a disease-free equilibrium point when infectives are recruited, which is in agreement with [29, 30]. A sensitivity analysis of the basic reproduction number indicates that rate of recruitment, death rate, transmission probability, and number of sexual partners of infected persons are the most sensitive parameters that can be used to control the spread of the disease. Thus, these parameters are those that should be targeted most by policymakers in the fight against the disease. Due to the International Labour Organization's campaign for no discrimination on the basis of ones' HIV status at the workplace, using the recruitment rate might be compromised, but using the other parameters can still be of immense help. The model is extended to an optimal control problem by incorporating time-varying controls into the basic model and the conditions for optimality are derived using the Pontryagin's Maximum Principle [26]. Finally, numerical simulations of the resulting control problem are carried out to determine the effectiveness of various combinations of the controls. It is revealed from the simulation of the control problem that the strategy that employs all the control efforts is most effective in the fight against the disease. Thus, there is the need for a multifaceted approach in the fight against the spread of HIV/AIDS.

## Figures and Tables

**Table 1:** Model parameter descriptions and values used for simulations.

Parameter	Parameter description	Value	Reference
*Q*	Rate of recruitment	100 People (Year)^−1^	
*π*	Fraction of subpopulations recruited	0.04	
*α*_*p*_	Rate at which Careless-Productive Susceptibles become Careful	0.4 (Year)^−1^	
*α*_*n*_	Rate at which Careless-Non-Productive Susceptibles become Careful	0.3 (Year)^−1^	
*θ*_*p*_	Rate at which Careless-Productive Infectives become Careful	0.6 (Year)^−1^	
*θ*_*n*_	Rate at which Careless-Non-Productive Infectives become Careful	0.5 (Year)^−1^	
*ρ*_1_	Rate at which Careful-Non-Productive Susceptibles become Productive.	0.6 (Year)^−1^	
*ρ*_2_	Rate at which Careless-Non-Productive Susceptibles become Productive.	0.4 (Year)^−1^	
*σ*_1_	Rate at which Careful-Productive Infectives lose their Productivity.	0.4 (Year)^−1^	
*σ*_2_	Rate at which Careless-Productive Infectives lose their Productivity	0.6 (Year)^−1^	
*β*	Contact rate between susceptibles and infectives	0.344 (People)^−1^	[4]
*τ*	Modification parameter due to careless behavior towards sex	1.2	
*δ*_1_	Rate of progression of Productive Infectives into AIDs	0.100 (Year)^−1^	[4]
*δ*_2_	Rate of progression of Nonproductive Infective into AIDs	0.100 (Year)^−1^	[4]
*δ*_3_	Rate of progression of Productive Infective into AIDs	0.100 (Year)^−1^	[4]
*δ*_4_	Rate of progression of Productive Infective into AIDs	0.100 (Year)^−1^	[4]
*μ*	Natural Death rate	0.020 (Year)^−1^	[4]
*ψ*	AIDs related death rate	1.000 (Year)^−1^	[4]

**Table 2:** Sensitivity indexes of *ℛ*_0_.

Parameter	Parameter description	Sensitivity index
*Q*	Rate of recruitment	+1.000
*β*	Contact rate between Susceptibles and Infectives	+1.000
*c*	Average number of sexual partners of an infective per unit time	+1.000
*δ*_1_	Rate of progression of Productive Infectives into AIDs	−0.460
*δ*_2_	Rate of progression of Nonproductive Infective into AIDs	−0.015
*δ*_3_	Rate of progression of Productive Infective into AIDs	−0.033
*δ*_4_	Rate of progression of Productive Infective into AIDs	−0.082
*μ*	Natural Death rate	−1.138
*σ*_1_	Rate at which Careful-Productive Infectives lose their Productivity	+0.000
*σ*_2_	Rate at which Careless-Productive Infectives lose their Productivity	−0.350
*τ*	Modification parameter due to careless behavior towards sex	+0.312
*θ*_*n*_	Rate at which Careless-Non-Productive Infectives become Careful	+0.180
*θ*_*p*_	Rate at which Careless-Productive Infectives become Careful	−0.102
